# Wound-Healing Promotion and Anti-Inflammatory Properties of Carvacrol Prodrugs/Hyaluronic Acid Formulations

**DOI:** 10.3390/pharmaceutics14071468

**Published:** 2022-07-14

**Authors:** Lisa Marinelli, Ivana Cacciatore, Erica Costantini, Marilisa Pia Dimmito, Federica Serra, Antonio Di Stefano, Marcella Reale

**Affiliations:** 1 Department of Pharmacy, “G. d’Annunzio” University of Chieti-Pescara, 66100 Chieti, Italy; l.marinelli@unich.it (L.M.); ivana.cacciatore@unich.it (I.C.); marilisa.dimmito@unich.it (M.P.D.); serra.federica07@gmail.com (F.S.); antonio.distefano@unich.it (A.D.S.); 2 Department of Medicine and Science of Aging, “G. d’Annunzio” University of Chieti-Pescara, 66100 Chieti, Italy; erica.costantini@unich.it; 3 Department of Innovative Technologies in Medicine and Dentistry, “G. d’Annunzio” University of Chieti-Pescara, 66100 Chieti, Italy

**Keywords:** antimicrobials, carvacrol prodrugs, hyaluronic acid formulations, wound healing

## Abstract

Background. Wound healing (WH) is a complex process involving several stages, such as hemostasis, inflammation, re-epithelialization, and remodeling. Many factors can impair WH, and different pharmacological approaches were studied to date, but the increase in antibiotic resistance, invasiveness, treatment duration, and high cost, have often hampered the resolution of the wound. In this study, we investigated the possible application of water-soluble carvacrol prodrugs (WSCPs) and hyaluronic acid (HA) and their formulations (WSCPs/HA) to improve WH and regulate the inflammatory response. Materials and methods. Firstly, the cytotoxicity of 0.1, 1 and 10 µg/mL of HA, WSCPs and WSCPs/HA formulations were evaluated on HaCaT cells and THP-1 cell lines. The ability of WSCPs/HA formulations to modulate wound repair was evaluated in an in vitro model of WH, using HaCaT cells at 6, 18, and 24 h. The expression of WH mediators, after wound closure was determined by qRT-PCR. Following, we polarized THP-1 cells in M1/M2-like macrophages and tested the anti-inflammatory properties of WSCPs/HA formulations. After, we tested the in vitro WH model for the effects of conditioned medium (CM) from M1/M2-like cells cultured in the presence of WSCPs/HA. Results. Results showed that WSCPs/HA formulations were able to significantly raise the wound closure rate, compared to the single constituents, promoting a complete wound closure after 18 h for WSCP1/HA (10 µg/mL) and after 24 h for WSCP2/HA (10 µg/mL), modulating the MMPs, TGFβ, and COX-2 gene expression. The effects of CM derived from M1/M2 polarized cells cultured in the presence of WSCPs/HA determined WH regulation, with a better ability of the WSCP2/HA formulation to modulate the time-dependent expression of reparative and inflammatory mediators. Conclusion. Our data underline the possible application of WSCPs/HA formulations as bioactive agents for the regulation of the wound repair process by the modulation of inflammatory and remodeling phases, affecting the activity of immune cells.

## 1. Introduction

Wounds, defined as an unnatural break, can be caused by damage with thermal or physical origin or as a consequence of a pathological condition. The wound healing (WH) process can vary according to the progress of the repair process, and distinguish between acute wounds, which tend to heal in 8–12 weeks, or chronic wounds that generally tend to reoccur and have a healing time extending beyond 12 weeks [1,2]. Treatment of acute and chronic wounds represents a persistent and increasing medical and economic problem in our healthcare system [3]. The need to facilitate complete and fast regeneration, led to a development in the field of tissue engineering and regenerative medicine, to identify new treatment strategies. The role of polymers in WH essentially is linked to their biocompatibility, biodegradability, non-toxicity, and structural properties. The use of polymeric materials is an option for wound repair, for the multiple benefits they offer, such as the ability to promote the production of growth factors, enhance cellular migration, promote angiogenesis, and inhibit bacterial invasiveness [2,4].

In the scenario, hyaluronic acid (HA), also known as hyaluronan, is one of the most abundant and well-known natural polymers.

HA is a natural biodegradable, biocompatible polymer with a wide range of applications in medical, pharmaceutical, cosmetic, and body repair technologies [5]. HA is extensively distributed in the human body, representing the chief constituent of the extracellular matrix of connective tissues; it plays a pivotal role in lubrication, cell differentiation, inflammation, and cell growth by binding to specific cell receptors, such as CD44 and RHAMM, and tool-like receptors 2 and 4 (TLR4) [6]. Chemically, HA is a natural polysaccharide derived by the combination of *N*-acetyl-d-glucosamine and d-glucuronic acid linked via a glycosidic bond [7]. The HA polysaccharide backbone becomes rigid in physiological conditions due to a series of bonds between the axial hydrogen atoms, and the equatorial side chains, which leads to a twisted ribbon structure called a “coiled structure”. The high number of sugar units composing the HA polyanionic structure allows the binding to water molecules that give HA a gluey quality, like gelatin and makes it one of the most suitable building blocks for drug delivery applications [8]. Several benefits can be attributed to HA, such as preserving joint lubrication, treating articular disorders, filling soft tissue wrinkles, and aiding WH without exerting side effects [9,10].

WH is a complex process involving several overlapping stages that include inflammation, formation of granulation tissue, re-epithelialization, matrix formation, and remodeling [9,10]. Several factors are involved in stimulating and coordinating cellular events that occur during WH and the recruitment of non-immune and immune cells, such as keratinocytes, fibroblasts, macrophages, and neutrophils, which are involved in the healing process and could negatively impact the regeneration process [11,12,13]. The involvement of HA in the WH process depends on its molecular weight. High molecular weight (HMW) HA displays anti-angiogenic and anti-inflammatory properties, whereas low molecular weight (LMW) HA (<1000 kDa) acts oppositely, being pro-inflammatory and pro-angiogenic, promoting healing and driving angiogenesis in the wound [14].

An extrinsic factor that may impact WH, retarding the integrity restoration of the body tissue is represented by infections; bacteria upregulate and prolong the activity of pro-inflammatory cytokines decelerating the healing process, suggesting the use of antimicrobial combinational therapy in the management of the healing process [15,16]. In the last decades, carvacrol has captivated much attention for its ability to inhibit the growth of bacteria despite possessing poor physicochemical properties and low chemical stability which limited its application as an antimicrobial medicine [17]. Recently, Marinelli et al. reported a series of water-soluble carvacrol prodrugs (WSCPs) exerting antimicrobial activity against a panel of gram-positive and -negative organisms with Minimal Inhibitory Concentration (MIC) values in the range of 32–256 µg/mL and a lack of toxicity towards HaCaT cells [18]; unfortunately, the most active prodrugs (WSCP1-2) suffered from rapid hydrolysis in simulated fluids and low plasma stability that could limit their clinical application after oral administration. To overcome these limitations, different formulations have been explored. WSCPs were loaded on montmorillonite [19], halloysite clay minerals [20], palygorskite and sepiolite [21], and HA [22]. Notably, in this last paper, WSCPs/hyaluronic acid formulations (WSCPs/HA) were fully characterized in terms of the viscoelastic properties by using different percentages of HA (0.5, 3, 6%).

In the present work, we aim to investigate the potential WH and anti-inflammatory properties of carvacrol prodrugs/hyaluronic acid formulations previously prepared [22]. The most promising formulations in terms of drug release and texture—composed of the 3% of HA and the 2% of WSPC1-2—were assayed on human keratinocytes from adult skin (HaCaT cells) and human monocytic leukemia cell line (THP-1) for future development as a wound dressing in topical applications.

## 2. Materials and Methods

### 2.1. Materials

Sodium hyaluronate from Cockscomb (MW min 350 kDa) was purchased from TCI Chemicals (Tamil Nadu, India). All other chemicals, reagents, and solvents were extra pure grade. WSCPs were synthesized using previously reported synthetic routes [18].

### 2.2. WSCPs/HA Formulations

WSCPs/HA formulations were obtained by hydration of powder HA (3% *w*/*w*) and mixed with WSCP1 or WSCP2 (2% *w*/*w*) using Milli-Q, yielding the HA_30_, as reported by Marinelli et al. WSCPs/HA formulations were prepared by dissolving WSCPs (20 mg) in 1 mL of Milli-Q water. To the obtained drug solution, the proper HA amount was slowly added. Structured systems exhibiting a viscoelastic nature were obtained, as confirmed by rheological measurements [22].

### 2.3. Cell Culture

Human keratinocytes cell lines from adult skin (HaCaT cells) purchased by Cell Line Service (CLS Eppelheim, Baden-Württemberg, Germany) were grown in Dulbecco’s modified Eagle’s medium (DMEM, Merck KGaA, Darmstadt, Germany), supplemented with 4.5 g/L glucose, 2 mM L-glutamine and 10% heat-inactivated fetal bovine serum (FBS) (Merck KGaA, Darmstadt, Germany).

The human monocytic leukemia cell line (THP-1) was purchased from the ATCC (Manassas, VA, USA) and maintained in RPMI 1640 (Merck KGaA, Darmstadt, Germany) added with 0.05 mM of 2-mercaptoethanol, 10% of FBS, glutamine (2 mM), penicillin (100 U/mL), and streptomycin (100 mg/mL) (Merck KGaA, Darmstadt, Germany). Both cells were cultured at 37 °C in a 5% CO_2_ humidified incubator (HeraCell 240i, Thermo-Fisher Scientific, Waltham, MA, USA).

### 2.4. Cytotoxicity Assay

HaCaT and THP-1 cells were seeded on 96 well-plates at a density equal to 1 × 10^5^ in 100 μL of growth media and incubated with HA, WSCP1, WSCP2, WSCP1/HA, and WSCP2/HA, at the concentration of 0.1, 1, and 10 μg/mL for 24 h. To assess cell viability, the LDH assay (CytoTox96^®^ Non-Radioactive Cytotoxicity Assay, Promega, Madison, WI, USA) was performed, following the manufacturer’s protocol. At the end of incubation, 50 μL of supernatant sample and lysis solution were mixed and incubated for 30 min at room temperature. Next, to finalize the reaction, 50 μL of stop solution was added to each well and the absorbance was measured at a 490 nm wavelength with the GloMax-Multi Detection System (Promega, Madison, WI, USA); 10% *v*/*v* of dimethyl sulfoxide (DMSO) was used as the positive control. All assays were performed in triplicate.

### 2.5. In Vitro WH Assay

HaCaT cells were seeded at a density of about 1.5 × 10^6^ cells/well (in a 6-well plate) in complete medium at 37 °C and 5% CO_2_ (*v*/*v*) and grown for 24 h to allow them to reach about 90% confluence. Before starting treatments, cells were serum-starved overnight. The monolayer of synchronized cells was gently scratched across the center of the well with a sterile pipette tip (Ø = 0.1 mm), and after scratching, cell debris was removed by washing with phosphate buffered saline (PBS, Merck KGaA, Darmstadt, Germany). Fresh medium containing 10% *v*/*v* of FBS and HA, WSCP1, WSCP2, WSCP1/HA, and WSCP2/HA, at 0.1, 1, and 10 μg/mL, were added to each well. Images of experiments conducted in triplicate were obtained from the same fields immediately after scratching (T0) and after 6, 18, and 24 h, using a Leica DMIL inverted microscope (Leica, Wetzlar, Germany) and analyzed using Leica LAS EZ Application Suite image analysis software by manually selecting the distance between the opposite edges in the wound region. Measurements were made at three different points in each image and averaged. Untreated scratched cells were considered as the control. Quantification of relative wound closure was performed according to Equation (1):

(1) $$\%\;\mathrm{cell}\text{-}\mathrm{free}\;\mathrm{area}=\frac{\left[\%\;\mathrm{cell}\text{-}\mathrm{free}\;\mathrm{scratched}\;\mathrm{area}\;\mathrm{at}\;T0-\%\;\mathrm{cell}\text{-}\mathrm{free}\;\mathrm{area}\;\mathrm{at}\;T6\text{-}18\text{-}24\right]}{\left[\%\;\mathrm{cell}\text{-}\mathrm{free}\;\mathrm{scratched}\;\mathrm{area}\;\mathrm{at}\;T0\right]}\times 100$$

### 2.6. THP-1 Monocytes Differentiation in Macrophages M0 and M1/M2 Polarization

Macrophage polarization is the process that leads to the pro-inflammatory M1 or proliferative M2 phenotype. For the establishment of the inflammatory model, 1.5 × 10^6^ THP-1 cells were seeded into 6-well culture plates, in presence of 1 mL of complete growth medium. Cells were differentiated into macrophage-like cells by adding 50 ng/mL of phorbol-12-myristate 13-acetate (PMA, Merck KGaA, Darmstadt, Germany) and, after 2 days, the PMA supplemented media was removed, and the resulting macrophages (M0) were activated for 6 h with 20 ng/mL of IFNγ and 1 μg/mL of LPS added to the fresh culture medium to obtain the M1 polarized macrophages or with 20 ng/mL of IL4 for 24 h to obtain the M2 polarized macrophages.

### 2.7. Treatment of M1/M2 Polarized THP-1 and Conditioned Media (CM) Collection

Macrophage-like cells M1 and M2 were incubated in a fresh growth medium at 37 °C, in the presence of 10 μg/mL HA, WSCP1, WSCP2, WSCP1/HA, and WSCP2/HA. After 24 h of incubation, samples were centrifuged at 1500 rpm for 5 min and the cell pellet and medium (conditioned medium CM) were collected.

### 2.8. RNA Extraction, Reverse Transcription (RT-PCR), and Real-Time PCR

Total RNA was extracted from HaCaT and THP-1 cells, after the culture treatments, using TRIzol reagent (Qiagen, Hilden, Germany). The RNA concentration was determined by measuring the absorbance of the samples (λ = 260 nm) using NanoDrop 2000 UV-Vis Spectrophotometer (Thermo Scientific, Waltham, MA, USA); its purity was assessed by the absorbance ratio of λ 260/280 nm and λ 260/230 nm. For each sample, 1 µg of RNA was reverse transcribed into complementary DNA (cDNA) using QuantiTect Reverse Transcription Kit (Qiagen, Hilden, Germany). A SYBR Green-based Real-Time PCR, with a melting curve analysis, was performed with GoTaq^®^ qPCR Master Mix (Promega, Madison, WI, USA) using the cDNA and specific primer pairs, to evaluate the gene expression of COX-2 (FW 5′-GACAGTCCACCAACTTACAATG-3′, RW 5′-GGCAATCATCAGGCACAGG-3′); TNF-α (FW 5′-CCTTCCTGATCGTGGCAG-3′, RW 5′- GCTTGAGGGTTTGCTACAAC-3′); MMP-2 (FW 5′-CAGTGACGGAAAGATGTGGT-3′, RW 5′-TGGTGTAGGTGTAAATGGGTG-3′); MMP-9 (Fw5′-GTCTTCCCCTTCACTTTCCTG-3′, RW 5′-GAGGAATGATCTAAGCCCAGC-3′); TGFβ (FW5′-AACAATTCCTGGCGATACCTC-3′, RW 5′-GTAGTGAACCCGTTGATGTCC-3′); IL-1β (FW 5′-TGAGGATGACTTGTTCTTTGAAG-3′, RW 5′-GTGGTGGTCGGAGATTCG-3′); MRC1 (FW 5′-ACCTGCGACAGTAAACGAGG-3′, RW 5′-GCTTGCAGTATGTCTCCGCT-3′); CD209 (FW5′-GTAGGACTGGATGTTGGGAAAT-3′, RW 5′-GAAAGAGAGAGAGGAGGAGGAA-3′); IL-10 (FW 5′-GAGAACCAAGACCCAGACATC-3′, RW 5′-TCACTCATGGCTTTGTAGATGC-3′) and VEGF (FW 5′-CCATGCCAAGTGGTCCCAGGC-3′, RW 5′-CGCATCGCATCAGGGGCACA-3′). For all the genes, the expression was normalized using the RPS18 (FW 5′-CTTTGCCATCACTGCCATTAAG-3′ RW 5′-TCCATCCTTTACATCCTTCTGTC-3′) gene expression levels.

All PCR reactions were performed in triplicate with CFX Real-Time PCR Detection Systems (Bio-Rad, Hercules, CA, USA), using the following conditions: initially, 2 min incubation at 95 °C followed by 40 cycles consisting of 30 s at 95 °C, then 1 min at 60 °C and 30 s at 68 °C. The analysis of the melting curve was performed in the temperature range of 60 to 95 °C at the end of each run. The relative expression of each gene was normalized by RPS18 using the ΔCT method, where ΔCT = CT (target gene)-CT (reference gene) [23]. Predicted cycle threshold values were directly exported into Excel worksheets for analysis. Relative changes in gene expression were determined by the 2^−ΔΔCT^ method, were ΔΔCt = ΔCt experimental sample-ΔCt calibrator and reported as the difference (n-fold) relative to the value for a calibrator sample (control = 1). Fold increases with respect to constitutive cells (mean ± SD) are representative of a total of three replicates performed for each experiment (*n* = 9).

### 2.9. Statistical Analysis

Data were summarized as the mean and standard deviation (SD) from three independent experiments. For real-time calculations with the 2^−ΔΔCt^ method, gene expression levels were reported as mean and 95% CI. Statistical comparison between the values from different treatments in the same cells model was calculated using GraphPad Prism software (http://www.graphpad.com; accessed on 1 December 2021) using a Student *t*-test for unpaired data. *p*-values were corrected for multiple comparisons when appropriate.

## 3. Results

### 3.1. Effects of HA, WSCPs, WSCPs/HA on the Viability of HaCaT and THP-1 Cells

**Figure 1:**
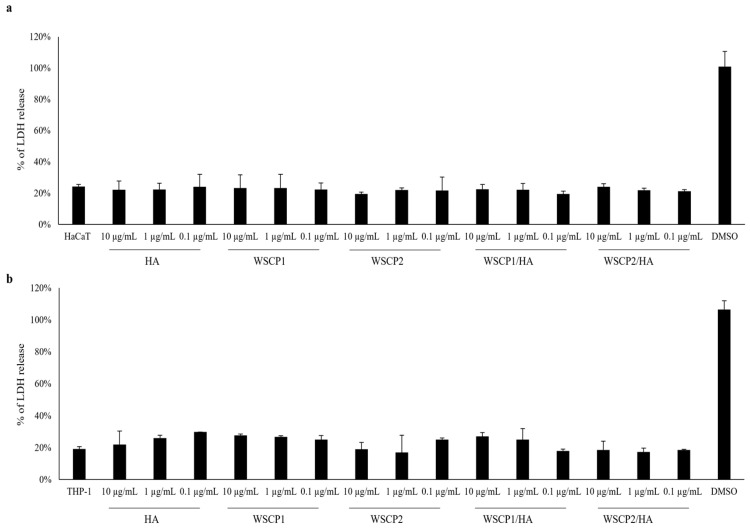
Cytotoxic effects of HA, WSCPs, and WSCPs/HA on HaCaT and THP-1 cells. (**a**) HaCaT cells were treated with 0.1, 1, and 10 µg/mL of HA, WSCP1, WSCP2, WSCP1/HA, and WSCP2/HA for 24 h. Percentage of the LDH release ± SD, for each treatment, were reported in relation to the DMSO assumed as 100% of cytotoxicity; (**b**) THP-1 cells were treated with 0.1, 1, and 10 µg/mL of HA, WSCP1, WSCP2, WSCP1/HA, and WSCP2/HA for 24 h. The percentage of the LDH release ± SD, for each treatment was reported assuming DMSO as 100% of cytotoxicity. Comparing treated cells vs. untreated cells, no significant differences were observed (Student *t*-test).

We performed preliminary experiments to rule out the cytotoxic effect of HA, WSCP1, WSCP2, WSCP1/HA, and WSCP2/HA at 0.1, 1, and 10 µg/mL by LDH assay. Results showed that both in HaCaT (Figure 1a) and THP-1 cells (Figure 1b), after 24 h of incubation in the presence of a different concentration of HA, WSCPs, and WSCPs/HA formulations, no significant change in the LDH levels was detected with respect to the untreated cells.

### 3.2. Effects of HA, WSCPs, and WSCPs/HA on the Re-Epithelialization of HaCaT Cell Line Scratched Monolayers

**Figure 2:**
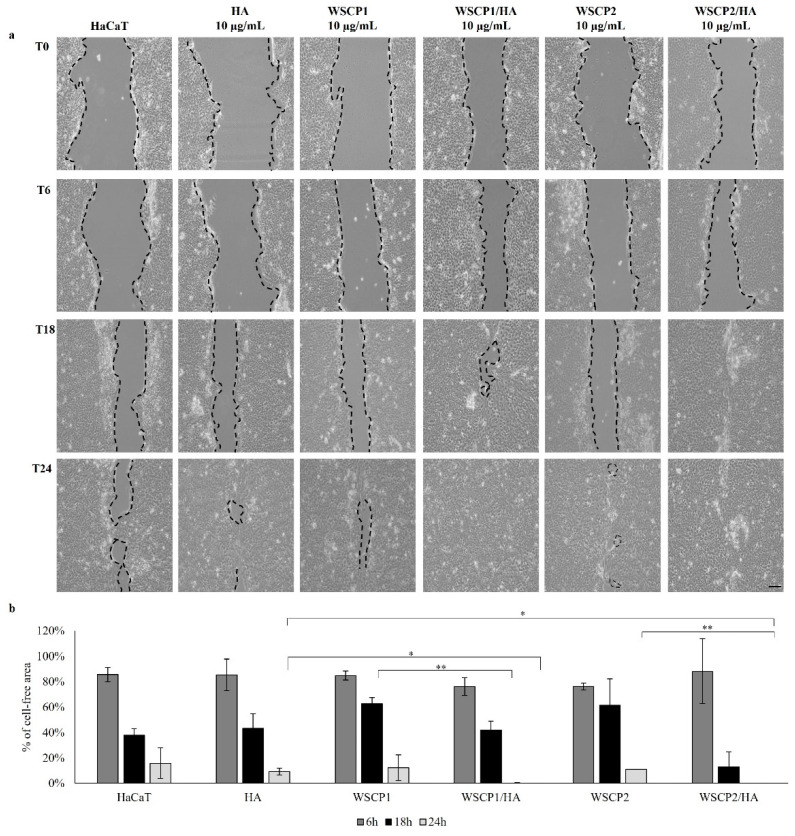
Effects of HA, WSCPs, and WSCPs/HA on HaCaT scratched cells. (**a**) Representative images of HaCaT monolayer re-epithelialization in the absence (control) or presence of HA, WSCP1, WSCP2, WSCP1/HA or WSCP2/HA (10 μg/mL) at 6, 18, and 24 h after injury (10× magnification). Scale bar: 10 µm. Fields show a representative of the width of quadruplicate wounds made in triplicate cultures. (**b**) Graphical representation of wound repair percentage in 10 μg/mL HA, WSCPs, and WSCPs/HA treated cells in comparison with their T0 (100% cell-free area), at a different time point (6, 18, and 24 h). Data are expressed as mean ± SD of three independent experiments. * *p* < 0.05; ** *p* < 0.01.

To understand the contribution of HA and WSCPs formulations to WH, the cell-based scratch assay with HaCaT cells monolayer was performed. Firstly, we evaluated over time (6, 18 and 24 h) the effects of different concentrations (0.1, 1, and 10 µg/mL) of HA; 10 µg/mL of HA significantly increased cell migration and wound closure, compared to the control group (43% cell-free area vs. 68% after 18 h and 9% vs. 16% cell-free area after 24 h) (Supplementary Material Figure S1). Additionally, the effects of WSCP1 and WSCP2, over time and at 0.1, 1, and 10 µg/mL were evaluated in scratched HaCaT cells. WSCP1 (10 µg/mL) induces 88% of the wound closure, and WSCP2 (10 µg/mL) induces 89% of the wound closure at T24 (24 h after a scratch), compared with T0 (100% cell-free area) (Supplementary Material Figure S2). Moreover, 0.1, 1, and 10 µg/mL of WSCP1/HA and WSCP2/HA formulations were analyzed over time in the WH assay. Observations with optical microscopy and images acquired at different time points showed that both WSCP1/HA and WSCP2/HA improve the wound closure compared to untreated scratched cells; 42% and 13% of cell-free area compared to 100% at T0, were observed at 18 h after scratching, in the presence of 10 μg/mL of WSCP1/HA and WSCP2/HA formulations, respectively. After 24 h, the complete closure of the wound was evident for both formulations with 0% of the cell-free area (Supplementary Material Figure S3). Thus, we have selected 10 μg/mL as the better concentration for the subsequent experiments. The effect of HA, WSCP1, WSCP2, WSCP1/HA and WSCP2/HA, at 10 μg/mL, was evaluated. As shown in Figure 2, no significant differences were detected at T6 while the main difference was observed after 18 h of incubation when the percentage of the cell-free area was significantly reduced in the presence of WSCP2/HA (13%), compared with the other formulations. After 24 h of incubation, both WSCP1/HA and WSCP2/HA drive the complete WH (100% of wound closure).

### 3.3. Effects of WSCP1/HA and WSCP2/HA Formulations on the Expression of WH Mediators

**Figure 3:**
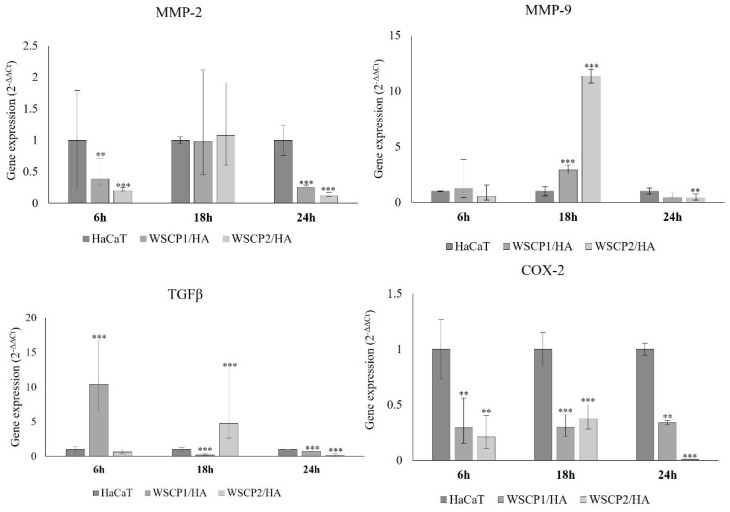
Gene expression in scratched HaCaT cells monolayer. Gene expression of MMP-2, MMP-9, TGFβ and COX-2 in scratched HaCaT cells monolayer treated with WSCP1/HA and WSCP2/HA (10 μg/mL). Changes in gene expression were determined by qRT-PCR, using the 2^−ΔΔCt^ method using RPS18 as a housekeeping gene. Data are reported as mean and 95% CI. ** *p* < 0.01; *** *p* < 0.001 vs. untreated HaCaT cells assumed as 1.

Matrix metalloproteinases (MMPs) are synthesized and secreted by keratinocytes and are essential for the remodeling of the pericellular microenvironment required for cell translocation and keratinocyte migration during WH [24]. The expression of MMP-2 and MMP-9 over time, during the WH in the presence of 10 µg/mL of WSCP1/HA or WSCP2/HA formulations was analyzed. After 18 h of incubation, the higher expression levels of MMP-2 and MMP-9 were observed with both WSCP1/HA and WSCP2/HA, with a higher MMP-9 increase. After 24 h of incubation, the levels of MMP-2 and MMP-9 decay significantly according to cell-free area reduction (Figure 3).

Transforming Growth Factor (TGF)β, which is chemotactic for fibroblasts, keratinocytes, and inflammatory cells plays an important role in the WH process. In agreement with the effects of WSCP1/HA and WSCP2/HA on WH, we observed a time-dependent effect of WSCP1/HA with an early increase in TGFβ expression at 6 h, and an up-regulation after WSCP2/HA treatment at 18 h of incubation (Figure 3). This suggested a different modulation of the WH mediator’s gene expression levels by the tested formulation, in line with the differential outcomes of wound re-epithelization. Moreover, the time-course of COX-2 gene expression during the WH of the scratched HaCaT cells monolayer in the presence of 10 µg/mL of WSCP1/HA and WSCP2/HA is reported. After 6, 18, and 24 h, COX-2 levels were down-regulated in the presence of both WSCP1/HA and WSCP2/HA, compared to the control.

### 3.4. Effects HA, WSCP-1/HA, and WSCP2/HA on Cytokine Expression in M1 and M2 Polarized THP-1 Cells

**Figure 4:**
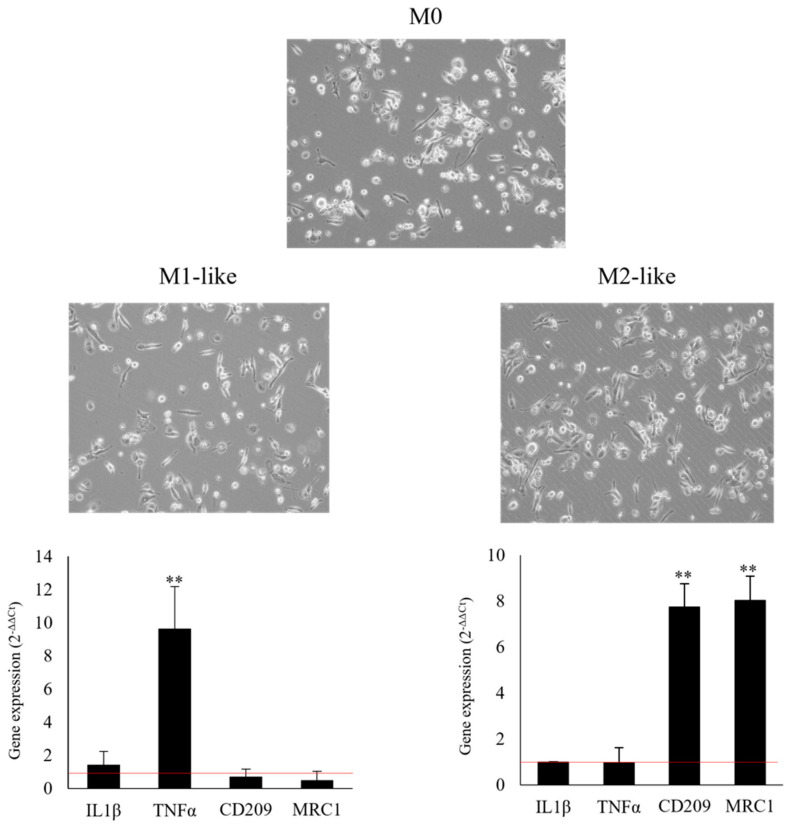
M1 and M2 polarization markers of THP-1 cells. Gene expression of IL-1β, TNFα (M1-polarization markers), CD290, and MRC1 (M2-polarization markers) in THP-1 polarized cells. Changes in cytokines gene expression were determined by qRT-PCR, using the 2^−ΔΔCt^ method, and RPS18 as a housekeeping gene. ** *p* < 0.01 vs. untreated M0 polarized cells assumed as 1 (red line).

To generate M1 and M2 macrophage-like phenotypes, we used THP-1 to ensure decreased variability compared to monocytes from different donors [25]. After THP-1 differentiation into M1 and M2 macrophage-like phenotypes, we investigated the specific polarity-related markers using gene expression analysis. Results showed the M1-polarization of THP-1 cells, with the overexpression of M1-polarity-related gene TNFα and IL-1β, compared with the M0 macrophages. Additionally, the M2-polarized THP-1 cells showed the overexpression of CD209 and MRC1 M2-related markers, in relation to the M0 cells (Figure 4).

To clarify if WSCPs/HA formulations can modulate the expression of TNFα, as a pro-inflammatory cytokine and IL-10, as an anti-inflammatory cytokine, M1 and M2 polarized cells were incubated with the previously selected concentration of 10 µg/mL of WSCPs/HA. Results showed that WSCP1/HA determines an increase in TNFα, although in a not significant way. WSCP2/HA-treatment of polarized M1 cells leads to a down-regulation of IL-10 and TNFα gene expressions, compared with M1 untreated cells. Otherwise, WSCP2/HA up-regulates both cytokines in M2 cells, with significant up-regulation of IL-10 with respect to M2 untreated cells (Table 1).

### 3.5. Conditioned Medium of M1/M2-like Polarized THP-1 Cultured with WSCPs/HA Effect on WH

**Figure 5:**
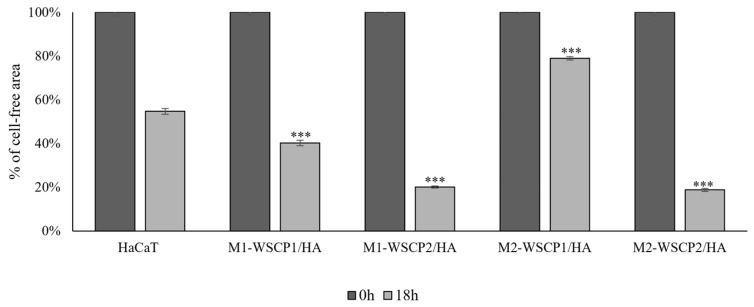
Effects of CM of M1/M2-like THP-1 in presence of HA, WSCPs, and WSCPs/HA on HaCaT scratched cells. Graphical representation of wound repair percentage in HA, WSCPs, and WSCPs/HA stimulated cells with respect to T0. Data are expressed as mean ± SD of three independent experiments. Statistically significant *p* values (*** *p* < 0.001) vs. untreated HaCaT cells.

Taking in mind that CM from polarized M1- or M2-like THP-1 cells in the presence of 10 µg/mL WSCP1/HA or WSCP2/HA, respectively, can mirror a microenvironment comparable to the inflammatory or proliferation phase of WH, it was added to the scratched HaCaT monolayer and the effects on the re-epithelialization were evaluated (Figure 5).

**Figure 6:**
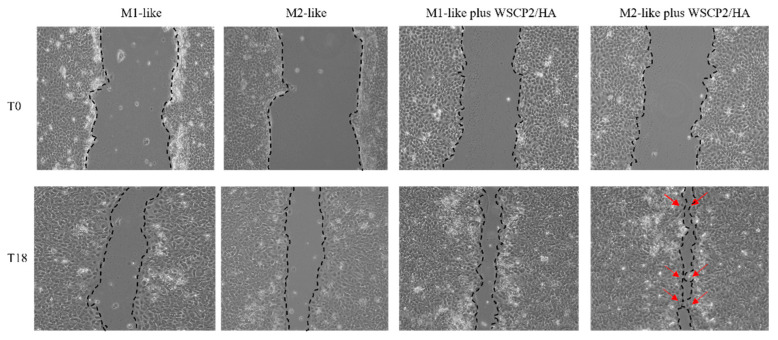
Effect of CM from M1/M2-like cells with or without WSCPs/HA on HaCaT scratch closure. Representative images of HaCaT scratched monolayer in presence of CM from M1-like, M2-like, M1-like plus WSCP2/HA and M2-like plus WSCP2/HA (10 μg/mL) CM (10× magnification). Red arrows indicate wound edge cells.

As reported in Figure 5, after 18 h of the mechanical wound, 54.6% of the cell-free area was observed in the untreated cells; in the presence of CM from M1 and M2 cells incubated with WSCP1/HA a cell-free area of 40.2% and 78.9%, respectively, was observed. In the presence of CM from M1 and M2 cells incubated with WSCP2/HA, the percentages of wound cell-free area were reduced to 20% and 18.7%, respectively. We highlight that WH may be differently driven by paracrine factors present in the CM of M1 or M2 polarized THP-1 cells incubated with WSCPs/HA. In fact, CM from M2-like cells incubated with WSCP2/HA influence the wound closure rate stimulating the wound edge to crawl towards the wound, and proximal cells could be activated in response to the movement of cells at the edge acting as a stimulus that can pull cells behind them in the same gap direction (Figure 6).

**Figure 7:**
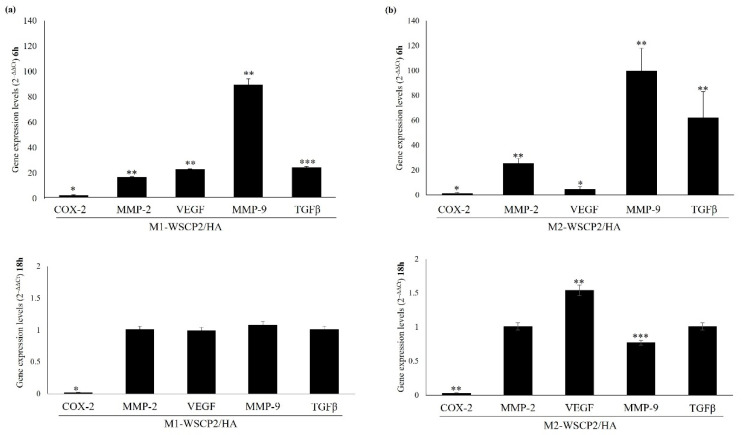
Gene expression in scratched HaCaT cells monolayer treated with CM obtained from M1- and M2-like cells cultured with WSCP2/HA. (**a**) Gene expression of COX-2, MMP-2, VEGF, MMP-9, and TGFβ in scratched HaCaT cells monolayer in presence of CM obtained from M1-like cells cultured with WSCP2/HA, for 6 and 18 h. Changes in gene expression were determined by qRT-PCR, using the 2^−ΔΔCt^ method. Data are reported as mean and 95% CI. * *p* < 0.05; ** *p* < 0.01; *** *p* < 0.001 vs. M1 untreated cells. (**b**) Gene expression of COX-2, MMP-2, VEGF, MMP-9, and TGFβ in scratched HaCaT cells monolayer in presence of CM obtained from M2-like cells cultured with WSCP2/HA, for 6 and 18 h. Changes in gene expression were determined by qRT-PCR, using the 2^−ΔΔCt^ method. Data are reported as mean and 95% CI. * *p* < 0.05; ** *p* < 0.01; *** *p* < 0.001 vs. M2 untreated cells.

Gene expression at 6 and 18 h after scratch, and in presence of CM from M1/M2-like cells incubated or not with WSCP2/HA were evaluated by qRT-PCR. Results indicated that after 18 h of incubation, both CMs down-regulate the early induced expression of MMP-2, MMP-9, COX-2, and TGFβ, with a stronger effect in the presence of CM from M2-WSCP2/HA. While VEGF is expressed in healed HaCaT-scratched cells, only in the presence of M2-WSCP2/HA (Figure 7).

## 4. Discussion

WH is an orchestrated process that is made up of overlapping phases with each regulating the progression toward the next one through the expression of soluble mediators, such as cytokines and degradative matrix metalloproteinases. Impaired WH is a result of the failure of normal progression through the phases [26]. In the last few years, an increasing number of reports have evaluated the effects of pharmacologic or physical modulation of tissue repair [27,28,29,30,31].

The main goal of this study was to evaluate the WH promotion and the anti-inflammatory activity of WSCPs/HA formulations. Innumerable HA-based drug delivery systems, such as nanoparticles, microneedle, and physical mixtures, have been developed to increase drug diffusion in skin diseases [32]. Most HA-based drug delivery systems exploit the physicochemical properties of HA responsible for its muco-adhesiveness, which allows the drug to remain absorbed at a specific action site, also modifying its release rate [33]. HA is appropriate as a biomaterial for tissue repair and suitable as a scaffold for holding cells, it is responsible for cell migration and proliferation, playing a key role in cell signaling mediating the organization of the extracellular matrix, and WH [34].

In this work, the effects of our formulations based on HA [22] were evaluated in an in vitro model of WH. Our results showed that these formulations can significantly raise the wound closure rate, compared to HA or WSCPs alone. In fact, WSCP1/HA and WSCP2/HA at 10 µg/mL induce complete healing after 24 h, with the earliest effect with WSCP2/HA, which after 18 h, led to the reduction of the cell-free area by up to 13%. These results are very interesting if considering the lower propensity to wound closure of HA and WSCPs when tested separately, suggesting that the formulation provides a synergic activity of both species which cooperate leading to the healing of the wound. Considering the effect of HA and WSCPs alone, this improved wound closure rate may be due to the HA stiffness in the solution which retains the WSCPs at the target site. Additionally, chemical hydrophilic interaction within the HA reticulum and the amino acid residues contained in WSCPs can stabilize and protect the active drug form from premature degradation improving the activity. Moreover, the hydrogel form of WSCPs/HA-dependent on the reticular structure of uncross-linked HA entraps the prodrugs, allowing the modulation of the drug release by an HA-concentration dependent diffusion mechanism.

In our experiments, we observed that treatment of HaCaT cells with WSCPs/HA for 18 h increased expression of both MMP-2 and MMP-9 with higher levels of MMP-9 in the presence of WSCP2/HA. TGFβ expression was higher in cells incubated with WSCP1/HA for 6 h, and with WSCP2/HA for 18 h. After 24 h, both formulations induced a similar trend of expression decline of MMP-2, MMP-9, and TGFβ, in accordance with the microscopic observation and measured distances, at 6 and 24 h, from the initial wound edge of the cells to the new edge, suggesting that WSCPs/HA formulations stimulate keratinocyte migration, driving the re-epithelization of the wound bed and later the complete wound closure. We can hypothesize that WSCPs/HA induced TGFβ, which acts as the WH promoting factor favoring keratinocyte migration and their protrusive activity [35]. The data from Wang et al. suggest that if TGFβ is present at the wound site for a long time, possibly due to its pro-inflammatory effect, WH is delayed and when in excess, TGFβ may prime to a hypertrophic scar [36]. Our data showed that at 24 h, the expression of TGFβ is not supported by WSCPs/HA, strengthening the hypothesis that WSCPs/HA promotes inflammation-proliferation-remodeling transition during WH.

Keeping in mind the role of pro-inflammatory M1 macrophages in an early phase of wound repair and later of the reparative M2 macrophages, and the ability of WSCP2/HA on accelerating WH, we have evaluated the effects of CM from M1 or M2 polarized THP-1 cells treated with WSCP2/HA.

The low levels of IL-10 expressed in M1 polarized cells incubated with WSCP2/HA could be responsible for the expression of mediators involved in the inflammatory phase of WH. The early increase in TGFβ expression in HaCaT treated with a CM from M1 polarized THP-1 cells treated with WSCP2/HA, is in accordance with its role in the initiation of inflammation, cell migration, and granulation tissue formation [37]. CM from M2 polarized THP-1 cells treated with WSCP2/HA significantly improved WH when added to a scratched monolayer of HaCaT cells, and significantly increased the expression of MMP-2, MMP-9, and TGFβ after 6 h; while after 24 h, a reduction of COX-2, MMPs, and TGFβ was detected. In M2-polarized THP-1 cells, WSCP2/HA induced a higher level of IL-10 that may be involved early in the expression of MMP-2 and MMP-9 and support the VEGF expression at 18 h. These results are in accordance with the hypothesized involvement of TGFβ in the regulation of VEGF and the downregulation of MMP-9 [38]. VEGF is one of the critical cytokines involved in WH, acting as a chemoattractant, and together with the promotion of keratinocyte migration by TGFβ, could lead to cell movements and play an important role at the wound site. Our results suggest that the effects of WSCPs/HA may depend on the regulation of overlapping phases of inflammation, regeneration (angiogenesis), and remodeling (fibrosis), supported by mediators produced by immune cells during WH.

**Figure 8:**
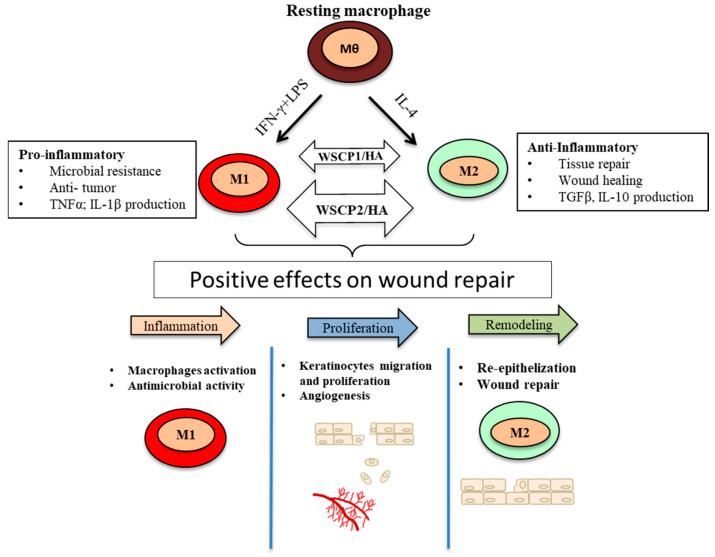
WSCP1/HA and WSCP2/HA promote wound repair through the activation of M1 and M2 macrophages. The M1 and M2 activation are mostly induced by WSCP2/HA formulations, driving the progression of WH from inflammatory to re-epithelization stages.

The effects of WSCP2/HA on WH and M1/M2 polarized THP-1 cells described above led us to hypothesize that WSCP2/HA may promote WH by modulating the expression of several mediators and the balanced signaling necessary to coordinate the phases transition during WH. Therefore, strengthening the reparative macrophages phenotype in wounds seems a particularly promising therapeutic approach (Figure 8). Understanding the role of developed WSCP2/HA formulation in molecular stages running to WH can help to respond to the growing need for new therapies for the treatment of impaired WH and improvement of clinical outcomes for patients with venous stasis ulcers, chronic pressure ulcers, diabetic ulcers, or massive traumatic wounds.

## 5. Conclusions

Polymer-based wound dressing material loaded with bioactive compounds can result in enhanced therapeutic outcomes when used in the treatment of wounds. Literature data reported that HA has been employed in the synthesis of different biological scaffolds for WH applications. In this paper, the important roles of HA-based formulations loaded with carvacrol prodrugs for skin WH were elaborated. They raise the wound closure rate by increasing the cell migration at the wound borders and modulating anti-inflammatory mediators. Moreover, the presence of WSCPs in prepared HA formulations could help to reduce bacterial invasion and support the treatment of infected wounds. Further studies will be conducted in vivo to better evaluate the involvement of WSCPs/HA formulations in the complex WH process.

## Figures and Tables

**Table 1:** Gene expression of anti-inflammatory and pro-inflammatory cytokines in M1 or M2 THP-1 polarized cells ^a^.

	IL-10	TNFα
M1-like	1 (1.64–0.60)	1 (2.70–0.36)
M2-like	1 (1.41–0.70)	1 (2.49–0.40)
M1-like plus WSCP1/HA	1.65 (4.57–0.57)	5.65 (15.92–2.00)
M2-like plus WSCP1/HA	1.49 (3.98–0.50)	1.01 (3.01–0.38)
M1-like plus WSCP2/HA	**0.22 (0.57–0.07)**	**0.04 (0.09–0.01)**
M2-like plus WSCP2/HA	**10.22 (28.70–3.62)**	1.73 (4.86–0.61)

^a^ Changes in TNFα and IL-10 gene expression were determined by qRT-PCR assay, using the 2^−ΔΔCt^ method and 18S as a housekeeping gene. Data reported as mean and 95% CI. Statistically significant *p* < 0.001 are shown in bold character.

## Data Availability

Not applicable.

## Supplementary Materials

The following supporting information can be downloaded at: www.mdpi.com/article/10.3390/pharmaceutics14071468/s1, Figure S1: Dose-Effect of HA on re-epithelialization of HaCaT cell line scratched monolayers at different time points; Figure S2: Dose-Effect of WSCP1 and WSCP2 on re-epithelialization of HaCaT cell line scratched monolayers at different time points; Figure S3: Effects of WSCPs/HA on re-epithelialization of HaCaT cell line scratched monolayers at different time points.
