# Supplementary figures and images for: Wound-Healing Promotion and Anti-Inflammatory Properties of Carvacrol Prodrugs/Hyaluronic Acid Formulations
Source: Pharmaceutics. 2022 Jul 14;14(7):1468. doi: 10.3390/pharmaceutics14071468 (PMC9323613; doi:10.3390/pharmaceutics14071468)

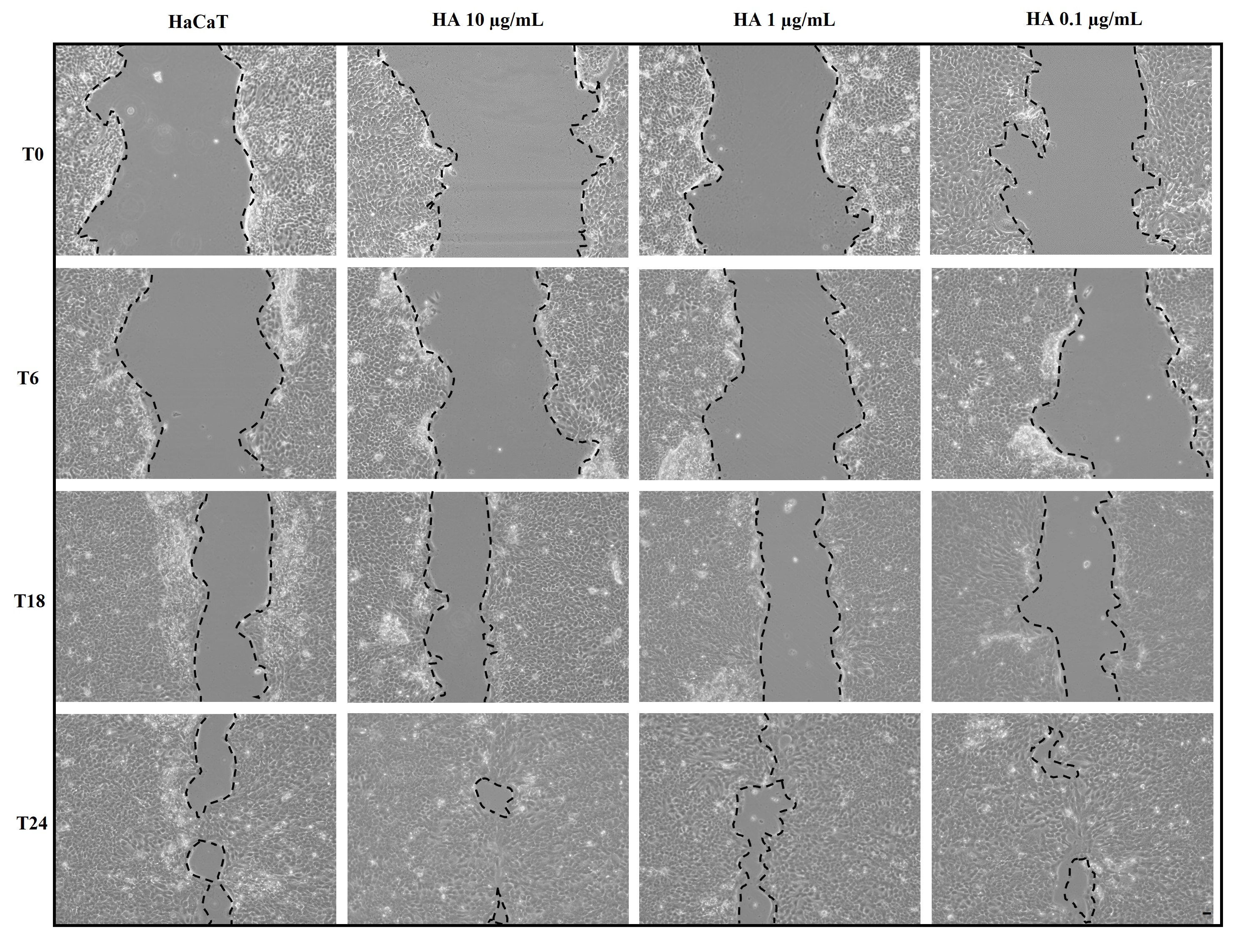

Supplement: Supplementary file 1 [file pharmaceutics-14-01468-s001.zip › pharmaceutics-1802826-supplementary/Figure S1.jpg]

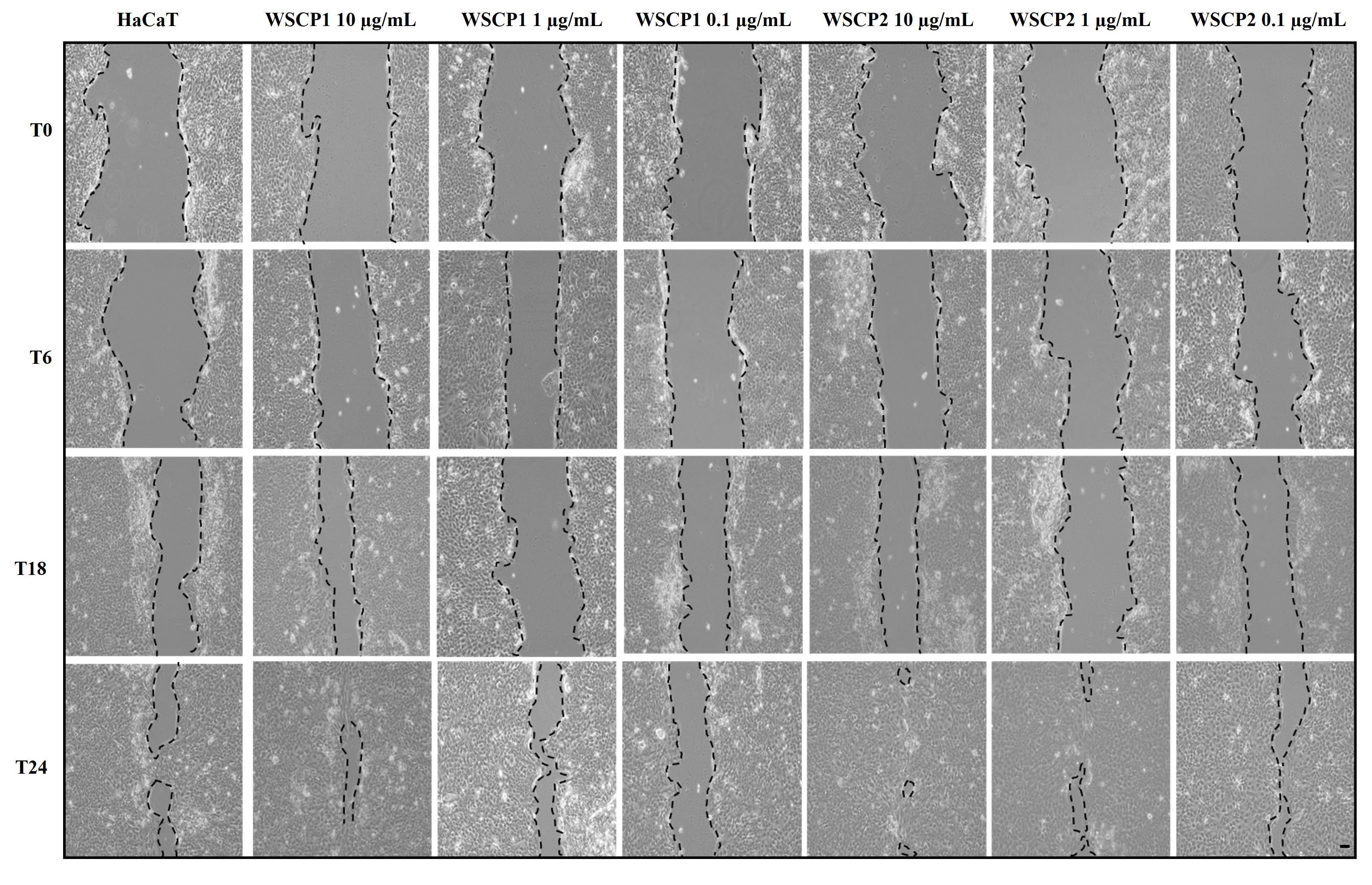

Supplement: Supplementary file 1 [file pharmaceutics-14-01468-s001.zip › pharmaceutics-1802826-supplementary/Figure S2.jpg]

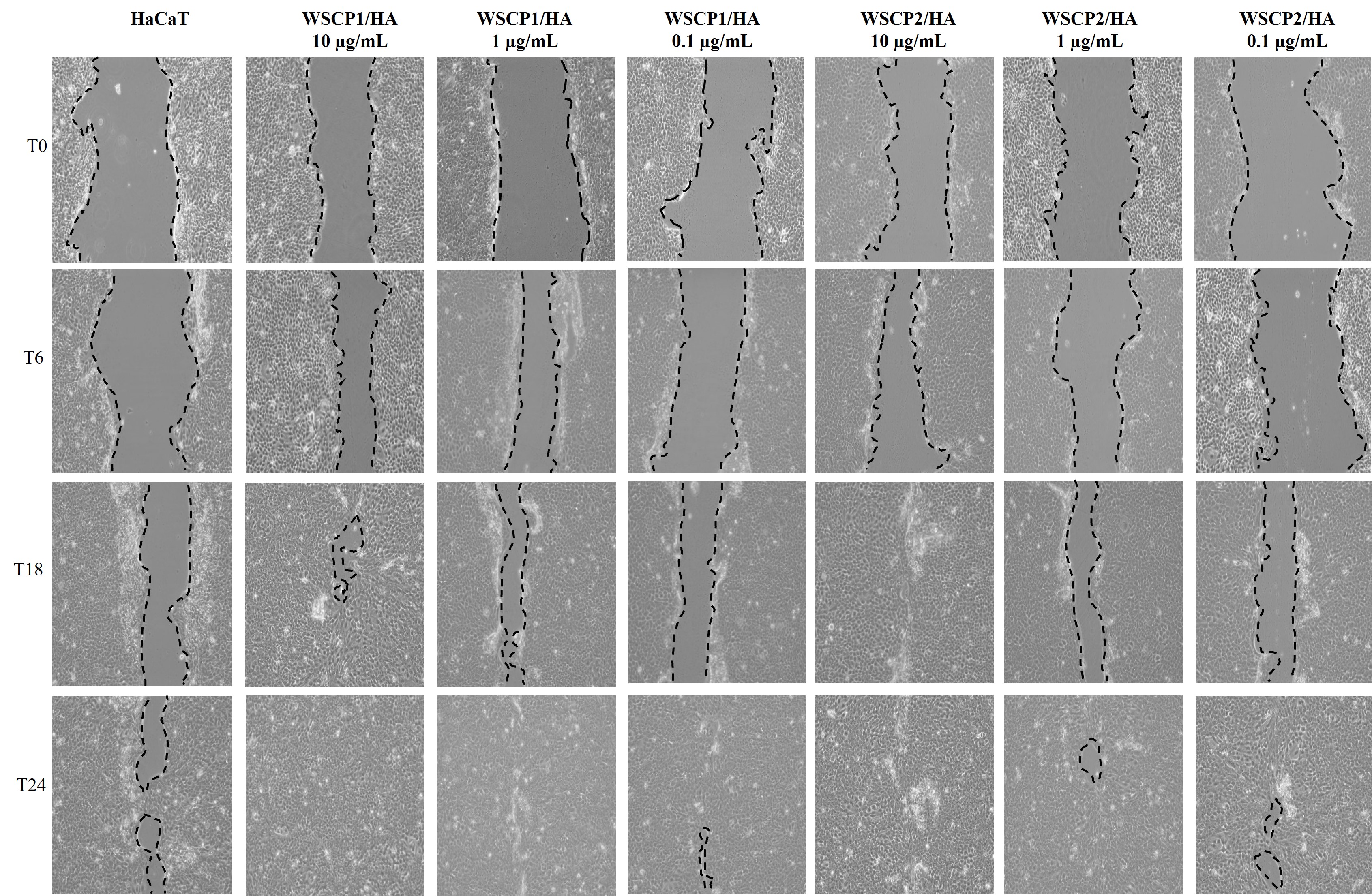

Supplement: Supplementary file 1 [file pharmaceutics-14-01468-s001.zip › pharmaceutics-1802826-supplementary/Figure S3.jpg]
